# A PNPLA3-Deficient iPSC-Derived Hepatocyte Screen Identifies Pathways to Potentially Reduce Steatosis in Metabolic Dysfunction-Associated Fatty Liver Disease

**DOI:** 10.3390/ijms25137277

**Published:** 2024-07-02

**Authors:** Caren Doueiry, Christiana S. Kappler, Carla Martinez-Morant, Stephen A. Duncan

**Affiliations:** 1 Department of Regenerative Medicine and Cell Biology, Medical University of South Carolina, Charleston, SC 29425, USA; doueiry@musc.edu (C.D.); morantc@musc.edu (C.M.-M.); 2 Medical Scientist Training Program, Medical University of South Carolina, Charleston, SC 29425, USA

**Keywords:** NAFLD, MAFLD, stem cells, steatosis, small molecules, drug discovery

## Abstract

The incidence of nonalcoholic fatty liver disease (NAFLD), or metabolic dysfunction-associated fatty liver disease (MAFLD), is increasing in adults and children. Unfortunately, effective pharmacological treatments remain unavailable. Single nucleotide polymorphisms (SNPs) in the patatin-like phospholipase domain-containing protein (PNPLA3 I148M) have the most significant genetic association with the disease at all stages of its progression. A roadblock to identifying potential treatments for PNPLA3-induced NAFLD is the lack of a human cell platform that recapitulates the PNPLA3 I148M-mediated onset of lipid accumulation. Hepatocyte-like cells were generated from *PNPLA3^−^*^/^*^−^* and *PNPLA3^I148M/M^*-induced pluripotent stem cells (iPSCs). Lipid levels were measured by staining with BODIPY 493/503 and were found to increase in *PNPLA3* variant iPSC-derived hepatocytes. A small-molecule screen identified multiple compounds that target Src/PI3K/Akt signaling and could eradicate lipid accumulation in these cells. We found that drugs currently in clinical trials for cancer treatment that target the same pathways also reduced lipid accumulation in PNPLA3 variant cells.

## 1. Introduction

Non-alcoholic fatty liver disease (NAFLD), also called metabolic dysfunction-associated fatty liver disease (MAFLD), is characterized by hepatic fat accumulation, which is not due to alcohol consumption. NAFLD is the most common chronic liver disease in the West and is rapidly increasing in both children and adults [1,2]. With time, NAFLD can progress from a less severe phenotype of steatosis to pathogenic nonalcoholic steatohepatitis (NASH), fibrosis and cirrhosis, and eventually hepatocellular carcinoma (HCC) [3,4]. NAFLD is expected to be the leading cause of end-stage liver disease and of hepatocellular carcinoma in the next decade [3]. Decompensated cirrhosis is the fourteenth most common cause of death in adults [5], and hepatocellular carcinoma is the third most common cause of cancer mortality [6]. NAFLD progression can increase mortality risk by 70% [3], but there are no effective steatosis treatment options besides diet and exercise [7]. 

Several etiologies contribute to NAFLD, including diet, the gut microbiota, epigenetics, drugs, and genetic variants [8]. According to genome-wide association studies (GWASs), a variant of the patatin-like phospholipase domain containing protein A3 (PNPLA3 (I148M)) has the most significant association with the disease and with hepatic triglyceride (TG) accumulation [9]. It also contributes to all steps of disease progression from steatosis to HCC [3]. The *PNPLA3 (I148M)* variant (rs738409) is formed by a nonsynonymous C to G substitution [10]. However, despite the strength of the genetic data, the molecular mechanisms underlying the contribution of the *PNPLA3 (I148M)* variant to NAFLD and its progression remain poorly defined.

PNPLA3 is highly expressed in the liver [11], is present on the surface of lipid droplets [12], and exhibits triglyceride lipase [13] and acylglycerol transacylase [14] activities. However, the variant protein does not appear to contribute to NAFLD through changes in its enzymatic function. Although PNPLA3 (I148M) loses 80% of its hydrolase activity [15], a loss-of-function mutant that specifically disrupts hydrolase activity did not result in lipid accumulation in mice [16]. Moreover, neither TG synthesis [17] nor phosphatidic acid turnover [18] were increased in the presence of the PNPLA3 variant in transgenic mice, indicating that an increase in acylglycerol transacylase activity is not responsible for the observed steatosis. 

It has been proposed that PNPLA3 (I148M) may evade ubiquitination, forming an abnormally stable interaction with lipid particles in hepatocytes, protecting them from proteolytic turnover and processing [9,17,18]. The lysine residues in the patatin domain and the C-terminal region of PNPLA3 are normally ubiquitinated and the protein is subsequently degraded by autophagy or proteasomes [9,17]. However, the PNPLA3 (I148M) variant is resistant to ubiquitination, leading to the accumulation of the protein on lipid droplets, with no changes in mRNA levels [17,18]. Moreover, the expression of recombinant isoforms of PNPLA3 that retained enzymatic activity but lacked lysine residues, and hence resisted ubiquitination, resulted in the accumulation of the protein and development of hepatic steatosis in a transgenic mouse model [9]. This implies that PNPLA3 (I148M) contributes to NAFLD through its accumulation on lipid droplets rather than through loss of its triglyceride hydrolase activity [9]. However, data concerning the mechanism through which PNPLA3 contributes to NAFLD are not conclusive and caveats include whether the mouse model accurately recapitulates the activity of PNPLA3 that is found in humans. While some studies suggest that PNPLA3 accumulation leads to reduced very-low-density lipoprotein triglyceride (VLDL-TG) secretion, others suggest that pathways such as triglyceride incorporation into VLDL, VLDL-TG secretion, and fatty acid oxidation are not affected [17].

A roadblock to identifying potential treatments for PNPLA3-induced NAFLD is the lack of a cellular platform that recapitulates *PNPLA3 (I148M)*-mediated onset of lipid accumulation in human hepatocytes. Most studies have been conducted in mouse models; however, the PNPLA3 protein in mice is shorter than in humans and predominantly localizes in adipose tissue rather than in the liver [9].

Recently, hepatocytes generated from human iPSCs containing either the PNPLA3 I148M variant or a PNPLA3 knockout allele were found to have increased lipid accumulation when treated with free fatty acids [19,20,21], which was consistent with the hypothesis that the PNPLA3 (I148M) phenotype is a consequence of loss-of-function. Such data suggest that differences exist between mouse and human models, which could affect the rational design of pharmaceuticals. 

We have previously used iPSC-derived hepatocytes as a platform to identify compounds for the potential treatment of a variety of genetic diseases that affect the liver [22,23,24,25,26,27]. In the current study, we used human PNPLA3 mutant iPSC–derived hepatocytes to effectively model the onset of NAFLD and provide a platform for the discovery of pathways involved in lipid accumulation in the variant background. We demonstrated that a series of small molecules and drugs, which are currently in clinical trials for the treatment of cancer, that target Src/PI3K/Akt could inhibit lipid accumulation in PNPLA3 variant hepatocytes.

## 2. Results

### 2.1. Generation of PNPLA3^∆1/∆2^ and PNPLA3^I148M/M^ iPSC-Derived Hepatocytes

**Figure 1:**
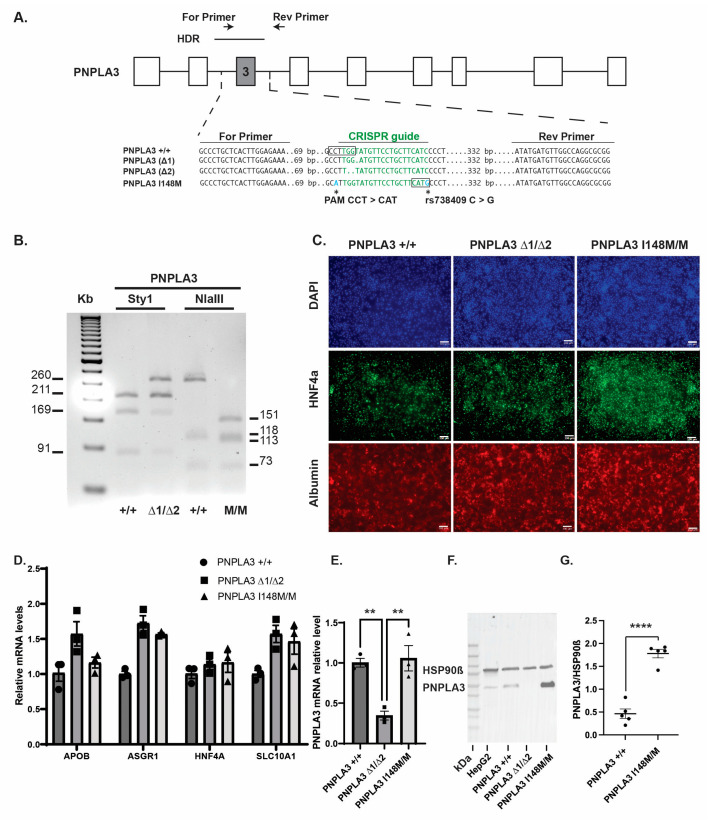
Generation of *PNPLA3^∆1/∆2^*, and *PNPLA3^I148M/M^* iPSC-derived hepatocytes. (**A**) Schematic illustration of *PNPLA3* locus showing the nucleotide sequences within exon 3 of the *PNPLA3* wildtype and mutated alleles; the CRISPR/Cas9 guide sequence (green); the relative positions of the primers used to identify the mutations and C > G substitution (rs738409); and the relative position of the HDR template and its sequence containing a silent substitution at the PAM site (blue). StyI and NlaIII cut sites are boxed. (**B**) Agarose gel showing PCR amplicons digested with Sty1 or NlaIII. The StyI recognition site is disrupted in the Δ2 indel resulting in a 260 bp band in *PNPLA3^∆1/∆2^* instead of the 91 and 169 bp bands in *PNPLA3^+/+^* cells. Residual 91 and 169 bp bands were detected in the *PNPLA3^∆1/∆2^* since the StyI cut site is not disrupted in the allele with the Δ1 indel. The C to G substitution in the *PNPLA3^I148M/M^* line creates an NlaIII recognition site, creating products of 151 bps and 113 bps in *PNPLA3^I148M/M^* cells compared to a 264 bp product in wildtype. (**C**) Immunostaining showing expression of hepatic markers HNF4a and Albumin in day 20 *PNPLA3^+/+^*, *PNPLA3^∆1/∆2^*, and *PNPLA3^I148M/M^* iPSC-derived hepatocytes compared to DAPI. Scale bar, 100 µm. (**D**) Bar graph showing the results of qRT-PCR for characteristic hepatocyte mRNAs in day 20 *PNPLA3^+/+^*, *PNPLA3^∆1/∆2^*, and *PNPLA3^I148M/M^* iPSC-derived hepatocytes (*n* = 3 biological replicates, mean ± SEM). (**E**) Bar graph showing the results of qRT-PCR of relative steady-state *PNPLA3* mRNA levels in *PNPLA3^+/+^, PNPLA3^∆1/∆2^*, and *PNPLA3^I148M/M^* iPSC-derived hepatocytes on day 20 (*n* = 3 biological replicates, mean ± SEM, ANOVA ** *p* ≤ 0.01, *PNPLA3^+/+^* vs. *PNPLA3^∆1/∆2^* CI [0.2180,1.097], and *PNPLA3^∆1/∆2^* vs. *PNPLA3^I148M/M^* CI [−1.152, −0.2730]). (**F**) Immunoblots using mouse monoclonal anti-ADPN (C-8) that specifically detects PNPLA3 in extracts from HepG2 cells, *PNPLA3^+/+^*, and *PNPLA3^I148M/M^*, but not in *PNPLA3^∆1/∆2^* iPSC-derived hepatocytes. (**G**) Quantification of immunoblots demonstrating a 3.8-fold increase in steady-state PNPLA3 I148M/M protein levels (*n* = 5 biological replicates, **** *p* ≤ 0.0001).

Tilson et al. previously demonstrated that when iPSC-derived hepatocytes with either a targeted disruption in *PNPLA3* or a *PNPLA3^I148M/M^* allele were treated with free fatty acids [20] they exhibited markedly elevated levels of cytoplasmic lipids compared to isogenic control cells. We first sought to confirm these findings using a genetically distinct population of iPSCs harboring PNPLA3 variants. We generated iPSCs with a PNPLA3 frameshift as well as the PNPLA3 I148M variant using CRISPR-Cas9 (Figure 1A). We chose to use genome engineering to introduce variations into K3 iPSCs [28] because this approach provided a series of isogenic iPSCs along with matched parental iPSCs. To produce loss-of-function cells, we designed a guide RNA sequence to target exon 3 of the *PNPLA3* gene because the locus is the site of SNP rs738409, which strongly correlates with NAFLD and encodes the PNPLA3 I148M variation [29]. The PAM site of the CRISPR guide lies adjacent to a StyI restriction enzyme cut site (Figure 1A). We reasoned that INDELs introduced into this locus would disrupt StyI cutting and, therefore, allow us to screen for deletions at the locus. Using this approach, we identified a compound heterozygous iPSC line with deletions of 1 bp in one allele and 2 bps in the other, resulting in frameshifts and the introduction of premature stop codons (p.[Val143AspfsTer26] and [Leu142PhefsTer28], referred to as *PNPLA3^∆1/∆2^*). We also synthesized a 466 bp homology-directed repair (HDR) template to introduce the rs738409 SNP into PNPLA3 to create the *PNPLA3^I148M/M^* line. The introduction of the SNP generated a novel NlaIII restriction enzyme cut site, and variants were detected using PCR, followed by restriction enzyme digestion of the exon 3 amplicon (Figure 1A,B). The genotype of the iPSC lines, including parental K3 cells, was confirmed by DNA and cDNA sequencing.

All iPSC lines expressed the pluripotency markers Nanog and octamer-binding transcription factor 4 (OCT4) and could differentiate into all three germ lineages in embryoid bodies (Supplementary Figure S1). We next confirmed that the control parental K3 iPSCs, as well as the *PNPLA3^∆1/∆2^* and *PNPLA3^I148M/M^* iPSCs, could similarly differentiate into hepatocyte-like cells, as described previously [30,31]. Immunostaining (Figure 1C) and qRT-PCR (Figure 1D, Supplementary Figure S1) revealed that characteristic hepatocyte markers [32] hepatocyte nuclear factor 4a (HNF4a), albumin, apolipoprotein B (ApoB), asialoglycoprotein receptor 1 (ASGR1), and solute carrier family 10 member 1 (SLC10A1) were expressed at comparable levels in iPSC-derived hepatocytes from each genotype, confirming that hepatocyte differentiation was similar between the lines. qRT-PCR revealed that *PNPLA3* mRNA levels were depleted by 70% in the *PNPLA3^Δ1/∆2^* hepatocytes compared to both the wild-type (*p* = 0.0089) and *PNPLA3^I148M/M^* hepatocytes (*p* = 0.006), likely due to nonsense-mediated RNA decay (Figure 1E) [33]. No change in *PNPLA3* RNA levels were identified in *PNPLA3^I148M/M^* iPSC-derived hepatocytes. Moreover, sequencing of cDNA derived from residual *PNPLA3* transcripts in *PNPLA3^Δ1/∆2^* iPSC-derived hepatocytes revealed the presence of the expected PNPLA3 frameshift sequences and no wild-type transcripts. Similarly, sequencing of cDNA derived from the transcripts of *PNPLA3^I148M/M^* iPSC-derived hepatocytes revealed a C to G substitution.

As discussed above, changes in PNPLA3 protein levels have been associated with the variants and so to determine whether protein levels were affected in PNPLA3^I148M/M^ iPSC-hepatocytes we sought to identify antibodies that specifically recognize the PNPLA3 protein [17,18]. We screened eight different commercial anti-PNPLA3 antibodies and found that ADPN Antibody (C8) and ADPN Antibody (D-5), detected a ~53 kDa protein in HepG2 and PNPLA3^+/+^ extracts that was absent from PNPLA3^∆1/∆2^ iPSC-hepatocytes (Figure 1F and Supplementary Figure S2). When the level of PNPLA3 was compared between all genotypes using the specific C8 antibody, PNPLA3 was found to be 3.8-fold higher in *PNPLA3^I148M/M^* compared to *PNPLA3^+/+^* iPSC-hepatocytes (*p* ≤ 0.0001, *n* = 5) (Figure 1G).

### 2.2. Both PNPLA3^I148M/M^ and PNPLA3^∆1/∆2^ iPSC-Derived Hepatocytes Display Increased Accumulation of Lipids 

**Figure 2:**
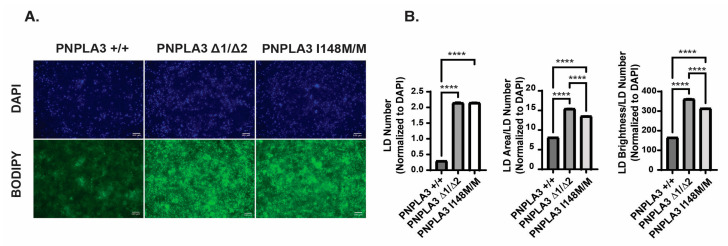
Lipid levels are elevated in both *PNPLA3^∆1/∆2^*, and *PNPLA3^I148M/M^* iPSC-derived hepatocytes. (**A**) Representative images of BODIPY 493/503 staining in *PNPLA3^+/+^*, *PNPLA3^∆1/∆2^*, and *PNPLA3^I148M/M^* iPSC-derived hepatocytes on day 20. DAPI staining was used to quantify cell numbers. Scale bar, 100 µm. (**B**) Quantification of BODIPY 493/503 staining in each of the cell lines (*n* = 89 *PNPLA3^+/+^*, *n* = 87 *PNPLA3^∆1/∆2^*, and *n* = 91 *PNPLA3^I148M/M^* biological replicates; mean ± SEM, ANOVA **** *p* ≤ 0.0001, normalized LD number in *PNPLA3^+/+^* vs. *PNPLA3^∆1/∆2^* CI [−1.922, −1.777], *PNPLA3^+/+^* vs. *PNPLA3^I148M/M^* CI [−1.924, −1.781], LD area/number in *PNPLA3^+/+^* vs. *PNPLA3^∆1/∆2^* CI [−7.628, −6.988], *PNPLA3^∆1/∆2^* vs. *PNPLA3^I148M/M^* CI [1.562, 2.195], *PNPLA3^+/+^* vs. *PNPLA3^I148M/M^* CI [−5.746, −5.113], LD brightness/number in *PNPLA3^+/+^* vs. *PNPLA3^∆1/∆2^* CI [−203.4, −187.9], *PNPLA3^∆1/∆2^* vs. *PNPLA3^I148M/M^* CI [39.71, 55.11], and *PNPLA3^+/+^* vs. *PNPLA3^I148M/M^* CI [−155.9, −140.5]). Lipid droplet number, area per droplet, and brightness per droplet were normalized to DAPI cell counts.

We predicted that both PNPLA3 variations would increase the accumulation of lipids within iPSC–hepatocytes; therefore, we measured lipid droplet (LD) number, size, and staining intensity using BODIPY 493/503 (Figure 2A) [34]. The *PNPLA3^I148M/M^* iPSC-derived hepatocytes showed a 10-fold increase in lipid droplet number on day 20 of differentiation compared to the control *PNPLA3^+/+^* iPSC-derived hepatocytes (*p* < 0.0001) (Figure 2A,B), while the intensity and size of the droplets increased by approximately two-fold. Similarly, *PNPLA3^∆1/∆2^* hepatocytes also increased lipid droplet number by 10-fold and droplet size and staining intensity by almost two-fold compared to control *PNPLA3^+/+^* hepatocytes (*p* < 0.0001). This increase in lipid accumulation is consistent with findings reported by others [19,20] and supports the view that loss of PNPLA3 function is associated with steatosis in patients. Importantly, these data suggest that the differentiation of *PNPLA3^∆1/∆2^* and *PNPLA3^I148M/M^* iPSC-derived hepatocytes could offer a platform to identify compounds with physiologically relevant lipid-lowering activities.

### 2.3. PNPLA3^∆1/∆2^ Hepatocytes Provide a Platform for Discovery of Small Molecules That Reduce Lipid Levels 

Currently, there are no approved treatments available for NAFLD other than the recommendations of diet and exercise [7]. The identification of therapeutics for NAFLD has also been complicated by the observation that PNPLA3 variants can reduce the efficacy of potential drugs, including the GLP-1 receptor agonist exenatide [35]. It may therefore be beneficial to use human PNPLA3 variant cells as an initial screening platform.

**Figure 3:**
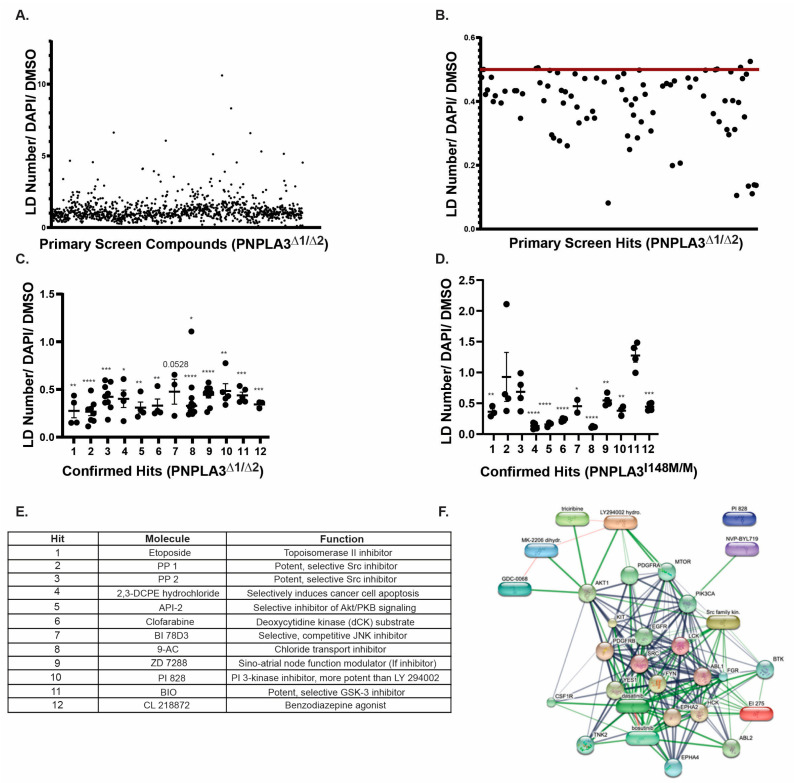
A small-molecule screen identifies compounds capable of reducing lipid droplets in both *PNPLA3^∆1/∆2^* and *PNPLA3^I148M^* iPSC-derived hepatocytes. (**A**) Graph showing results of the primary screen. Lipid droplet number was normalized to DAPI and compared to the average DMSO lipid droplet number/DAPI within the plate. (**B**) Graph showing data excerpted from the primary screen for compounds reducing lipid droplet content ≥50% compared to DMSO. The red line shows a 50% reduction in lipid droplet content compared to DMSO. (**C**) Graph showing the reproducibility of primary hits in *PNPLA3^∆1/∆2^* cells (*n* ≥ 3, mean ± SEM, Student’s *t*-test, * *p* ≤ 0.05, ** *p* ≤ 0.01, *** *p* ≤ 0.001, **** *p* ≤ 0.0001). (**D**) Graph showing the reproducibility of confirmed hits in *PNPLA3^I148M/M^* cells (*n* ≥ 2, mean ± SEM, Student’s *t*-test, * *p* ≤ 0.05, ** *p* ≤ 0.01, *** *p* ≤ 0.001, **** *p* ≤ 0.0001). (**E**) Table listing compounds and their functions. Compound numbers correspond to those used in graphs (**C**) and (**D**). (**F**) STITCH analyses of compounds (lozenges) and interacting proteins (spheres) to predict functional networks affected by compounds that reduce lipid accumulation in *PNPLA3^∆1/∆2^* and *PNPLA3^I148M/M^* iPSC-derived hepatocytes.

To identify potentially druggable pathways that could reduce the accumulation of lipids in hepatocytes harboring PNPLA3 variants *PNPLA3^∆1/∆2^* iPSCs were seeded in 96-well plates and differentiated into hepatocyte-like cells. The cells were treated with 5 µM each of the 1120 biologically active compounds from the Tocriscreen Mini library from days 15 to 20 of differentiation when lipid droplet accumulation normally begins, and we were able to reproducibly measure increased lipid droplet levels in the PNPLA3 variant cells (Supplementary Figure S3). We chose this specific library because each of the compounds have a known mechanism of action and can be used in an unbiased way to identify critical pathways involved in lipid regulation. After five days of treatment, the cells were stained with BODIPY 495/503 and lipid droplet content was assessed. Each plate included 16 wells treated with DMSO, which served as vehicle-treated control wells. For the primary screen, we used an automated image capture Keyence microscope and quantified both lipid droplet numbers by BODIPY 495/503 staining and cell numbers by staining the nuclei with DAPI. The number of lipid droplets in each well was normalized to DAPI and then to the average number of lipid droplets per cell in DMSO (vehicle) wells. We defined primary hits of interest as compounds that could reduce the number of lipid droplets by at least 50% compared to DMSO. The primary screening identified 86 compounds (Figure 3A,B). To determine which hits were reproducible, these 86 compounds were tested again at 5 µM in independent differentiations using a minimum of three replicate wells. Of the initial hits, we confirmed 12 compounds that caused a significant and reproducible lowering of lipids in *PNPLA3^∆1/∆2^* iPSC-hepatocytes (*p* ≤ 0.05) (Figure 3C). 

We expected that any bona fide lipid-reducing compound would have a similar impact on lipid levels in *PNPLA3^I148M/M^* hepatocytes. Of the 12 initial compounds identified in the primary screen using *PNPLA3^∆1/∆2^* iPSC-hepatocytes, nine also caused a reduction of ≥50% (*p* ≤ 0.05) in the number of lipid droplets in *PNPLA3^I148M/M^* hepatocytes compared to vehicle treatment (Figure 3D). The glycogen synthase kinase 3 (GSK-3) inhibitor 6-bromoindirubin-3′-oxime (BIO) had no observable effect on *PNPLA3^I148M/M^* iPSC-hepatocytes, despite reducing lipid levels in *PNPLA3^∆1/∆2^* iPSCs; therefore, it was excluded. Treatment with Src kinase inhibitors 4-amino-5-(4-methylphenyl)-7-(t-butyl)pyrazolo[3,4-d]-pyrimidine (PP1) and 4-amino-5-(4-chlorophenyl)-7-(t-butyl)pyrazolo[3,4-d]-pyrimidine PP2 tended toward significance (*p* = 0.9093 and 0.1935, respectively) but displayed variation between replicate wells. Although we noted this variability, we retained Src kinase inhibitors as hits because of their robust lipid-lowering effects in *PNPLA3^∆1/∆2^* cells. The identity of each of the compounds is shown in Figure 3E. Of the 11 initial hits, we excluded compounds from further studies if their defined targets were not reported to be expressed in the liver. These compounds were 2,3-DCPE hydrochloride, clofarabine, 9-AC, ZD 7288, and CL 218872. 

### 2.4. Src, PI3 Kinase, or Akt Inhibition Can Reduce Lipid Accumulation in PNPLA3-Depleted iPSC-Hepatocytes 

We reasoned that a compound’s ability to impact lipid levels would be more likely to be bona fide if multiple compounds were identified that targeted interacting pathways. Therefore, we performed bioinformatics analyses using STITCH and found that inhibitors of Src, PI3K, and Akt formed an interactome (Figure 3F) [36].

We purchased new aliquots of each compound targeting Src, PI3K, and Akt to reduce the possibility of artifacts originating from the library. We also rationalized that if multiple compounds with shared modes of action yielded similar outcomes, it would increase the confidence in our conclusions. Therefore, we added the PI3K inhibitor LY 294002 and the Akt inhibitor MK-2206, which were not present in the original library. 

**Figure 4:**
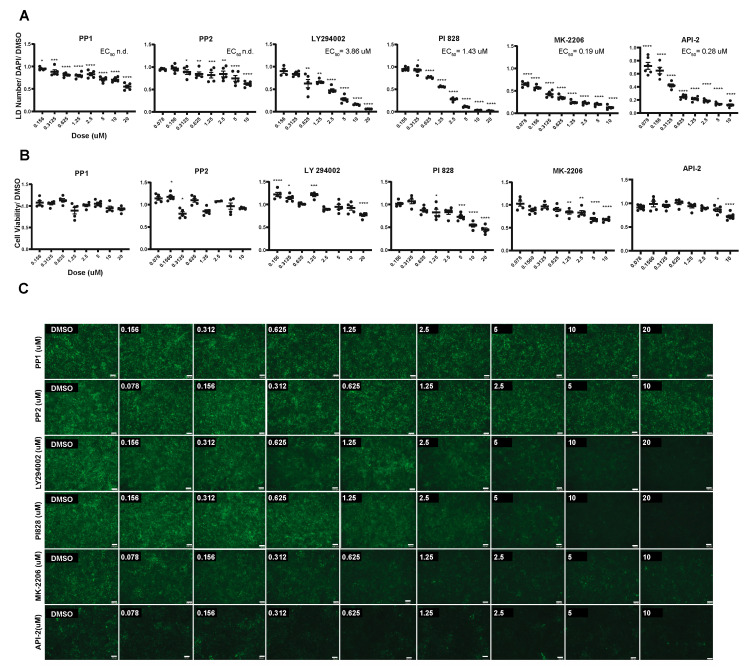
Inhibitors of Src, PI3K, or Akt can reduce lipid levels in *PNPLA3^∆1/∆2^* iPSC-derived hepatocytes. (**A**) Graphs showing the results of the dose-response assays assessing lipid droplet accumulation in *PNPLA3^∆1/∆2^* with increasing concentrations of inhibitors compared to DMSO (*n* ≥ 4, mean ± SEM, ANOVA compared to DMSO, * *p* ≤ 0.05, ** *p* ≤ 0.01, *** *p* ≤ 0.001, and **** *p* ≤ 0.0001). (**B**) Graphs showing cell viability of *PNPLA3^∆1/∆2^* cells treated with different concentrations of inhibitors (*n* ≥ 2, mean ± SEM, ANOVA compared to DMSO, * *p* ≤ 0.05, ** *p* ≤ 0.01, *** *p* ≤ 0.001, and **** *p* ≤ 0.0001). (**C**) Representative images showing BODIPY 493/503 staining in *PNPLA3^∆1/∆2^* iPSC-derived hepatocytes after treatment with DMSO or increasing concentrations of inhibitors, which are shown within the insets. Scale bar, 100 μm.

Multiple inhibitors targeting each pathway were then tested in eight-point dose-response assays on hepatocytes derived from *PNPLA3^∆1/∆2^* (PP1, PP2, LY294002, PI828, MK-2206, and API-2; Figure 4A–C and Supplementary Figure S4). A representative subset of compounds that targeted each pathway (PP1, LY294002, and MK-2206; Supplementary Figure S5) was also tested in *PNPLA3^I148M/M^* iPSC-derived hepatocytes. All tested compounds reduced the number of lipid droplets in both cell genotypes, although their efficacies varied. Inhibition of Src by inhibitors PP1 and PP2 reduced droplet levels only modestly by approximately 50%. In contrast, LY294002 and PI828, which are inhibitors of PI3K, and Akt inhibitors MK-2206 and API-2 almost eradicated the presence of lipid droplets (Figure 4A,C). We also tested the effect of the compounds on cell viability to ensure that any decrease in lipid droplet accumulation was not due to cell death. As expected, at high doses, most compounds affected cell viability, albeit modestly (Figure 4B). However, each inhibitor also effectively reduced lipid droplet accumulation at doses that had no significant impact on cell survival (Figure 4B). Based on these data, we conclude that inhibition of Src, PI3K, and Akt signaling reduces lipid accumulation in human PNPLA3 variant iPSC-derived hepatocytes. 

### 2.5. Cancer Drugs Are Candidates for Reducing Lipid Droplet Accumulation in the Presence of PNPLA3 Mutations 

**Figure 5:**
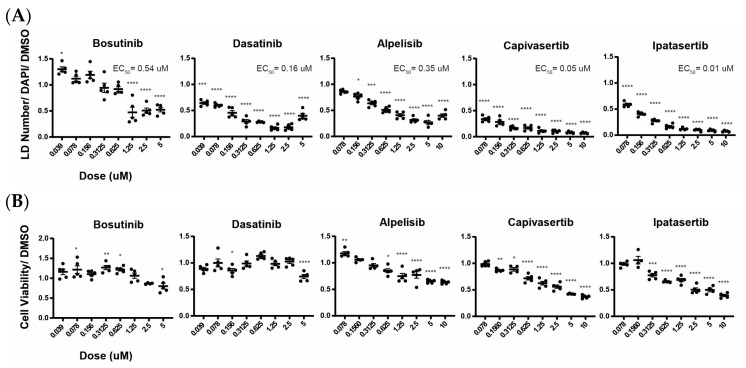
Cancer drugs targeting Src, PI3K, or Akt reduce lipid droplet accumulation in *PNPLA3^∆1/∆2^* iPSC-derived hepatocytes. (**A**) Graphs displaying the results of the dose–response assays assessing lipid droplet accumulation in *PNPLA3^∆1/∆2^* with increasing concentrations of cancer drugs targeting Src, PI3K, or Akt (*n* ≥ 3, mean ± SEM, ANOVA compared to DMSO, * *p* ≤ 0.05, *** *p* ≤ 0.001, and **** *p* ≤ 0.0001). (**B**) Graphs showing cell viability of *PNPLA3^∆1/∆2^* cells treated with different concentrations of cancer drugs in clinical trials (*n* ≥ 3, mean ± SEM, ANOVA compared to DMSO, * *p* ≤ 0.05, ** *p* ≤ 0.01, *** *p* ≤ 0.001, and **** *p* ≤ 0.0001).

Src, PI3K, and Akt signaling promote cancer cell growth and proliferation, and considerable effort has been devoted to generating pharmaceuticals targeting these pathways. Bosutinib and Dasatinib are Src inhibitors that are used to treat chronic myelogenous leukemia. Alpelisib is a PI3K inhibitor used for the treatment of metastatic breast cancer. Ipatasertib and Capivasertib are Akt inhibitors that are used in clinical trials for the treatment of a variety of aggressive cancers [37,38]. Since we confirmed that inhibiting Src, PI3K, or Akt can reduce lipid accumulation in the presence of PNPLA3 mutations, we next investigated whether these drugs have the potential to reduce lipid content in steatotic hepatocytes by performing eight-point dose–response assays on *PNPLA3^∆1/∆2^* iPSC-derived hepatocytes (Figure 5A and Supplementary Figures S4 and S6). All the drugs tested revealed substantial inhibition of lipid droplet accumulation and were effective even at nanomolar concentrations. We also tested the effects of these doses on cell viability (Figure 5B). As cancer drugs, these compounds are known to be effective, and unsurprisingly, treatment with high concentrations causes a decline in cell numbers. However, treatment with nanomolar concentrations of the drugs had little effect on cell survival, while still effectively reducing lipid droplet levels by 50–80%.

## 3. Discussion

We used *PNPLA3^∆1/∆2^* and *PNPLA3^I148M/M^* iPSC-derived hepatocytes as novel platforms for small-molecule screening and pathway identification. When successfully differentiated into hepatocyte-like cells, both genotypes showed increased lipid accumulation compared to wild-type iPSC-derived hepatocytes. These observations are in accordance with those of Tilson et al. [20]. We hypothesize that more stable I148M PNPLA3 protein accumulation is accompanied by a loss of triglyceride lipase function resulting in lower specific activity. Our screen showed that inhibitors of Src, PI3K, and Akt can reduce lipid accumulation in a dose-dependent manner when steatosis is induced by PNPLA3 mutations. Several studies have implicated PI3K and Akt in lipid accumulation, and some studies have suggested that Src regulates the PI3K/mTOR pathway by directly or indirectly activating the PI3K regulatory subunit [39,40]. Yang et al. showed that quercetin, a natural product that inhibits PI3K, decreases hepatic lipid accumulation in mouse models of diabetes-induced NAFLD, possibly through the regulation of bile acid homeostasis [41]. LY294002 also reduces lipid accumulation in goose livers owing to the signaling role PI3K plays in de novo lipogenesis and fatty acid oxidation [42]. One study revealed that inhibiting the PI3K catalytic subunit in mouse adipocytes can promote lipolysis and upregulate genes related to mitochondrial metabolic processes [43]. It is also important to mention that other studies show that PI3K inhibition can lead to hyperglycemia which could reduce enthusiasm for using inhibitors therapeutically [44]. The interactions between PI3K, insulin signaling, and glucose levels and how they impact pathological lipid levels is complex, and so any inhibition of PI3K in the setting of PNPLA3 mutations would need rigorous investigation. In addition to the role PI3K plays, Akt also regulates lipid metabolism through the mammalian target of rapamycin complex 1 (mTORC1) and SREBP, both of which increase lipid accumulation [45]. These data, along with our current findings, suggest that the PI3K/Akt pathway regulates lipid metabolism and is possibly associated with variants associated with NAFLD. 

Parafati et al. conducted a screening of AstraZeneca’s chemogenic library using a human induced pluripotent stem cell model of hepatocytes treated with fatty acids [46]. Their hits elucidated the cyclin D3-cyclin-dependent kinase 2–4 (CDK2-4)/CCAAT-enhancer-binding proteins (C/EBP)/diacylglycerol acyltransferase 2 (DGAT2) pathway as contributing to endoplasmic reticulum stress-induced steatosis. It is of note that DGAT2 is involved in SREBP-1c mediated lipogenesis [47]. Suppression of diacylglycerol acyltransferase-2 (DGAT2), but not DGAT1, with antisense oligonucleotides reversed diet-induced hepatic steatosis and insulin resistance, suggesting that PNPLA3-mediated lipid accumulation is possibly associated with DGAT2 signaling. 

Because our data show that inhibiting Src/PI3K/Akt can reduce hepatic lipid accumulation in the presence of PNPLA3 loss of function, we aimed to determine if drugs that are currently used to inhibit these pathways are also effective. Bosutinib and Dasatinib are FDA-approved Src inhibitors that are used to treat chronic myelogenous leukemia [38,48]. Alpelisib is a PI3K inhibitor approved for use in the PIK3CA-related overgrowth spectrum (PROS) [49]. Capivasertib and Ipatasertib are Akt inhibitors that are currently in clinical trials for the treatment of breast cancer [37,50]. Treatment of *PNPLA3^∆1/∆2^* hepatocytes with these drugs resulted in at least a 50% reduction in lipid accumulation compared to DMSO-treated *PNPLA3^∆1/∆2^* hepatocytes. Importantly, they were effective even at doses that did not impact cell viability and, except for Alpelisib, the half maximal effective concentration (EC_50_) for viability was higher than the EC_50_ for lipid reduction. While the specific drugs tested were developed for cancer treatment and have not been tested for chronic treatment of NAFLD, our data suggest that compatible pharmaceuticals that target the same regulatory pathways could be successful in reducing hepatic lipid accumulation in patients with PNPLA3 variants. These data also further confirm the role of Src/PI3K/Akt pathways in PNPLA3-mediated lipid accumulation 

Our screen identifies drugs that can reduce lipid accumulation after it has already begun on Day 15. If we were to translate this information to the clinical setting, it suggests that these drugs could contribute to a reduction in maximal lipid accumulation in NAFLD patients. Whether they can reverse steatosis after it has been chronically established remains to be tested, ideally using mice repopulated with *PNPLA3^I148M/M^* human hepatocytes, as has been described by others [51]. We suspect that early intervention using drugs that target the Src/PI3K/Akt pathways, may be more beneficial than during chronic lipid accumulation. 

While our study identifies the Src/PI3K/Akt pathway involvement in PNPLA3-related lipid accumulation, further studies are needed to understand how the pathway contributes to lipid accumulation with the loss of function of PNPLA3. In addition, understanding the mechanism of action of the small molecules and drugs used can provide a deeper understanding of the mechanism involved in reducing lipid accumulation in the presence of the PNPLA3 variant, both in vitro and in vivo. 

## 4. Materials and Methods

### 4.1. CRISPR/Cas9 Genome Editing 

For the PNPLA3 knockout, a CRISPR guide RNA sequence (TGGTATGTTCCTGCTTCATC) targeting exon 3 of *PNPLA3* was designed and cloned into the PX459 pSPCas9(BB)-2A-Puro plasmid (Ran et al., 2013). The plasmid was then introduced into iPSCs using Viafect (Promega, Madison, WI, USA, #E4981), then plated on Matrigel (Gibco, Waltham, MA, USA, #A1413302) to generate the *PNPLA3^∆1/∆2^* iPSCs. The guide RNA target site is adjacent to a StyI cutting site, and introduced INDELs would disrupt StyI cutting, allowing us to screen for deletions at the locus. To generate *PNPLA3^I148M/M^*, a 466 bp HDR template sequence was also designed, which contained exon 3 of *PNPLA3* harboring a C to G substitution that introduces the I148M mutation. The template also contained a silent C to A substitution to disrupt the PAM site. The C to G substitution introduces a new NlaII restriction site that allows diagnostic screening. The plasmid was made linear and the sequence was introduced into iPSCs plated on Matrigel along with the CRISPR guide to generate *PNPLA3^I148M/M^*. In both cases, transfected cells were treated with 1 ug/mL of Puromycin (Sigma, Burlington, MA, USA, P9620) for 2 days to select for colonies that received the introduced DNA. Resulting colonies were collected, and their genomic DNA extracted using QuickExtract™ DNA Extraction Solution (LGC Biosearch Technologies, Hoddesdon, UK, #QE09050). The DNA from each colony was used to run a Polymerase Chain Reaction with primers designed to amplify the targeted region using Herculase Fusion Polymerase (Agilent, Santa Clara, CA, USA, #600675) (For: GCCCTGCTCACTTGGAGAAA, Rev: ATATGATGTTGGCCAGGCGCGG). The amplification was followed by a diagnostic digest with the appropriate restriction enzyme and run on an agarose gel to determine which colonies had the required genotype. The genotypes of the candidate colonies were then confirmed by DNA and cDNA sequencing. 

### 4.2. Induced Pluripotent Stem Cell Culture and Differentiation 

All cells were routinely confirmed to be free of mycoplasma. Human male K3 iPSCs were generated from foreskin fibroblasts (ATCC CRL2097) and their detailed characterization, karyotyping, and short tandem repeat (STR) analyses has been previously described [28]. iPSCs were cultured in mTeSR medium [52,53] with 40 ng/ml of zebrafish basic fibroblast growth factor on an E-cadherin-IgG Fc fusion protein matrix [54] in 4% O_2_-5% CO_2_ incubators. At 24 h before differentiation, K3 cells were seeded as a monolayer on Matrigel (2 mg/mL)-coated tissue culture plates. The cells were induced to differentiate to hepatocyte-like cells using an established protocol [30,31,55]. Briefly, during the first 2 days of differentiation, the cells were cultured in RPMI 1640 Medium (Invitrogen, Waltham, MA, USA, #22400105) and supplemented with 2% B27 Supplement without insulin (Invitrogen, MA, USA, #A1895601), 100 ng/mL of Activin A (Invitrogen, MA, USA, #PHC9563), 20 ng/mL of Fibroblast Growth Factor 2 (FGF2) (Invitrogen, MA, USA, #PHG0023), and 10 ng/mL of BMP4 (Invitrogen, MA, #PHC9533). During the next 3 days, the cells received RPMI supplemented with B27 Supplement without insulin and 100 ng/mL Activin A. At that stage the cells had been induced to form definitive endoderm. During the next 5 days, the cells received RPMI supplemented with B27 Supplement containing insulin, 10 ng/mL FGF2, and 20 ng/mL BMP4 to convert them from definitive endoderm to hepatic progenitor cells. The cells then received an additional 5 days of RPMI supplemented with B27 Supplement and 20 ng/mL of Hepatocyte Growth Factor (Invitrogen, MA, USA, #PHG0321) to generate immature hepatocytes. Finally, the cells were cultured in HCM medium (Lonza, Walkersville, MD, USA, #CC3198) and supplemented with 20 ng/mL of Oncostatin M (Invitrogen, MA, USA, #PHC5015) during the last 5 days of differentiation, inducing them to become hepatocyte-like cells.

### 4.3. Small-Molecule Screen and Compound Library 

The Tocriscreen Mini library consists of 1120 biologically active compounds in DMSO that target defined cellular processes and pathways (Biotechne, Minneapolis, MN, USA, #2890). The library was validated previously by screening for developmental regulators of hepatocyte differentiation in iPSCs [23]. To conduct the primary screen, *PNPLA3^∆1/∆2^* iPSCs were plated for differentiation on 96-well plates. From day 15 to day 20 of differentiation, 16 wells of each plate were treated with either 0.9% DMSO or 5 µM of each compound/0.9% DMSO from the library. To determine reproducibility, the effects of compounds identified in the primary screen were repeated in iPSC-derived hepatocytes from both *PNPLA3^∆1/∆2^* and *PNPLA3^I148M/M^* cells. In these follow up assays, all wells were treated with compounds at a final concentration of 5 µM in 0.5% DMSO to reduce the background interference of DMSO in the assay. For dose–response curves, DMSO was reduced further to 0.1% DMSO. On day 21, the cells in all cases were stained with BODIPY 495/503 (Invitrogen, MA, USA, #D3922) to quantify lipid droplets and staining intensity along with DAPI to determine cell number. KEYENCE analysis software (BZ-X800 analyzer) was used to characterize lipid droplets per cell number. 

### 4.4. Cell Viability Assay 

A CellTiter-Glo^®^ luminescent cell viability assay kit (Promega, Madison, WI, USA, #G7572) was used to study the viability of cells treated with compounds. Compound-treated cells were tested at the same time as their DMSO-treated controls within the same plate. The assay was conducted using the manufacturer’s instructions, and luminescence was read with a SYNERGY/HTX multi-mode plate reader (BioTek, Winooski, VT, USA, #S1LFA).

### 4.5. Immunostaining 

To stain hepatocytes for characteristic hepatocyte markers, differentiated iPSCs were fixed in 4% paraformaldehyde (PFA) for 20 min at room temperature. Cells were permeabilized with 0.4% Triton-X-100 for 20 min at room temperature before being treated with 1% bovine serum albumin in PBS for one hour at room temperature and incubated with primary antibodies (ALB, 1:2000, Proteintech, Rosemont, IL, USA, #16475-1-AP; HNF4a, 1:2000, Santa Cruz, TX, USA, #SC-6556) at 4 °C overnight. Samples were washed 3 times with PBS for 5 min each and then incubated with secondary antibodies (Alexa Fluor 488 donkey anti-goat, 1:1000, Invitrogen, MA, USA, #A32814; Alexa Fluor 594 donkey anti-rabbit, 1:1000, Invitrogen, MA, USA, #A32754). Cells were washed 3 times with PBS and stained with DAPI (1 μg/mL). Images were taken using a KEYENCE BZ-800. For BODIPY staining, cells were washed with PBS and then stained with BODIPY 493/503 for 15 min in the dark at 37 °C after 20 days of differentiation. Cells were then washed twice with PBS, fixed with 4% paraformaldehyde, washed 3 times with PBS for 5 min each and stained with DAPI. The cells were imaged within 24 h, with 4 random images taken per well, which covered the majority of the well. The same image settings were used for compound-treated and DMSO-treated wells. Images were taken using KEYENCE BZ-800. Integrated fluorescent number, area, and brightness were normalized by total cell counts, as determined by DAPI staining.

### 4.6. Quantitative Real-Time PCR Analysis

RNA was isolated from *PNPLA3^+/+^*, *PNPLA3^∆1/∆2^*, and *PNPLA3^I148M/M^* hepatocytes using the Quick-RNA Mini kit (Zymo Research, Tustin, CA, USA, #R1055). First-strand cDNA was produced using M-MLV Reverse Transcriptase (ThermoFisher/Invitrogen, Waltham, MA, USA, #28025-013). TaqMan polymerase was used for quantitative real-time PCR, which was run on a BioRad CFX384 real-time PCR machine. TaqMan primer sequences are listed in Supplementary Table S1. 

### 4.7. Statistical Analysis

Biological replicates were defined as independent treatments with compounds performed on hepatocytes derived from iPSCs in independent wells of a tissue culture plate(s). Technical replicates were defined as repeated assays on a single sample. All graphs represent the means with the standard errors of the means. ANOVAs and Student’s *t*-tests were used depending on the data sets and the number of replicate wells, as specified in the figure legends. A primary hit was defined as a mean change in lipid droplet number of ≥50%. To confirm the reproducibility of each hit, Student’s *t*-tests were conducted. Statistical analyses were performed using GraphPad Prism version 9. The EC_50_ of each compound was determined using the Quest Graph EC50 Calculator (https://www.aatbio.com/tools/ec50-calculator, accessed on 28 June 2024). 

## 5. Conclusions

We generated hepatocyte-like cells from *PNPLA3^−/−^* and *PNPLA3^I148M/M^*-induced pluripotent stem cells. Our data show that the *PNPLA3^I148M/M^* variant causes lipid accumulation due to loss of function of the protein. Using our hepatocytes in a small-molecule screen identifies multiple compounds that target Src/PI3K/Akt signaling and that could eradicate lipid accumulation in these cells. We also showed that other drugs, currently in clinical trials for cancer treatment, also reduced lipid accumulation in our cells by targeting the same pathway. 

## Data Availability

All data, reagents and constructs will be shared upon request. In some cases, the MUSC may require completion of an MTA. Further information and requests for resources and reagents should be directed to and will be fulfilled by the corresponding author, Stephen Duncan: duncanst@musc.edu.

## Supplementary Materials

The following supporting information can be downloaded at https://www.mdpi.com/article/10.3390/ijms25137277/s1.
