# Supplementary material for: A PNPLA3-Deficient iPSC-Derived Hepatocyte Screen Identifies Pathways to Potentially Reduce Steatosis in Metabolic Dysfunction-Associated Fatty Liver Disease
Source: Int J Mol Sci. 2024 Jul 2;25(13):7277. doi: 10.3390/ijms25137277 (PMC11242544; doi:10.3390/ijms25137277)
Supplement: Supplementary file 1 [file ijms-25-07277-s001.zip › Supplementary Table S1.pdf]

Supplementary Table S1. Sequence of primers used for RT-PCR.

Gene	Forward Primer Sequence	Reverse Primer Sequence	Probe sequence
ApoB	CATTGCCCTTCCT CGTCTT	CCAGAGACAGA AGAA GCCAAG	CTGGATACCGTGTAT GGAAACT GCTCC
ASGR1	TCCTTTCTGAGCC ATTGCC	TGAAGTCGCTAG AGTCCCAG	CGTGAAGCAGTTCGT GTCTGACCT
SLC10A1	TGTACAGGAGGA GAGGCATC	ACCTGTCCAATG TCTTCAGTC	AACCTCAGCATTGTG ATGACCACCT
HNF4A	CTCATAGCTTGAC CTTCGAGTG	GGTGGACAAAG ACAAGAGGAA	TCTGGACGGCTTCCT TCTTCATGC
PNPLA3	GACATCACCAAGC TCAGTCTA	CATCCAAATATC CTCGAAGGCA	CTCTCCCAGCACCTT GAGATCCG
RPL13A	GCCTTCACAGCGT ACGA	CGAAGATGGCG GAGGTG	AGCAGTACCTGTTTA GCCACGATGG
