# Supplementary figures and images for: A PNPLA3-Deficient iPSC-Derived Hepatocyte Screen Identifies Pathways to Potentially Reduce Steatosis in Metabolic Dysfunction-Associated Fatty Liver Disease
Source: Int J Mol Sci. 2024 Jul 2;25(13):7277. doi: 10.3390/ijms25137277 (PMC11242544; doi:10.3390/ijms25137277)

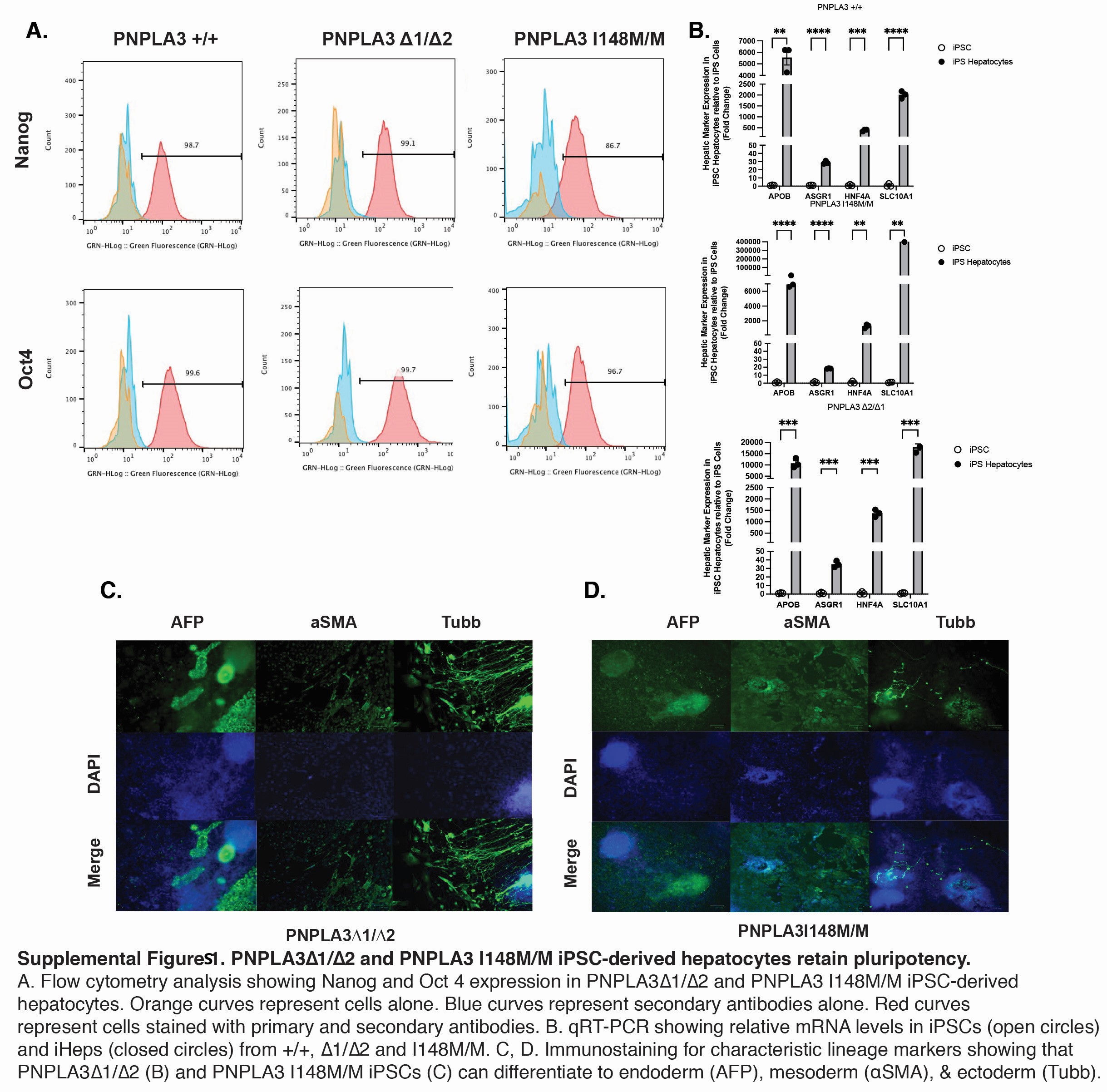

Supplement: Supplementary file 1 [file ijms-25-07277-s001.zip › Figure S1.jpg]

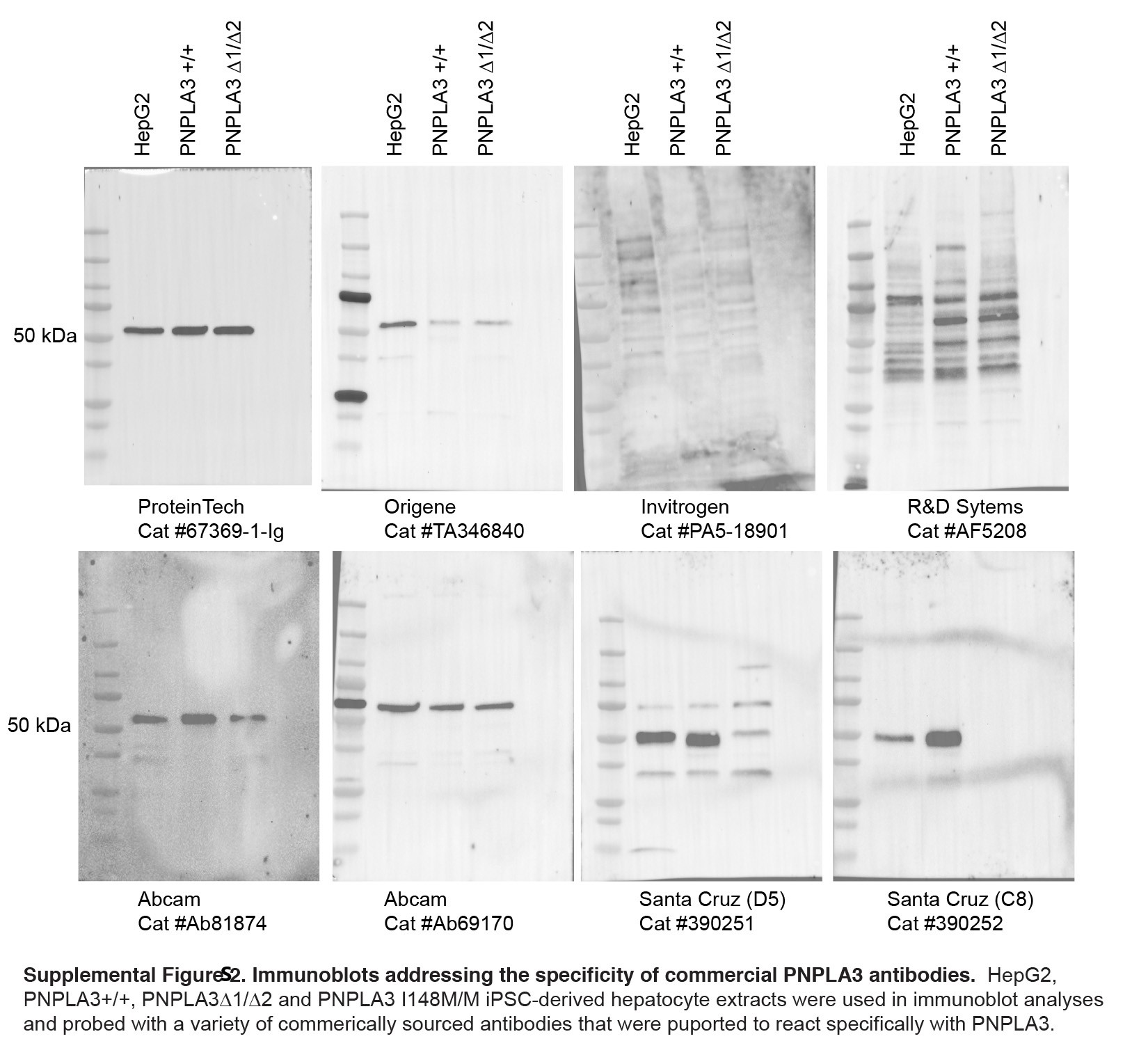

Supplement: Supplementary file 1 [file ijms-25-07277-s001.zip › Figure S2.jpg]

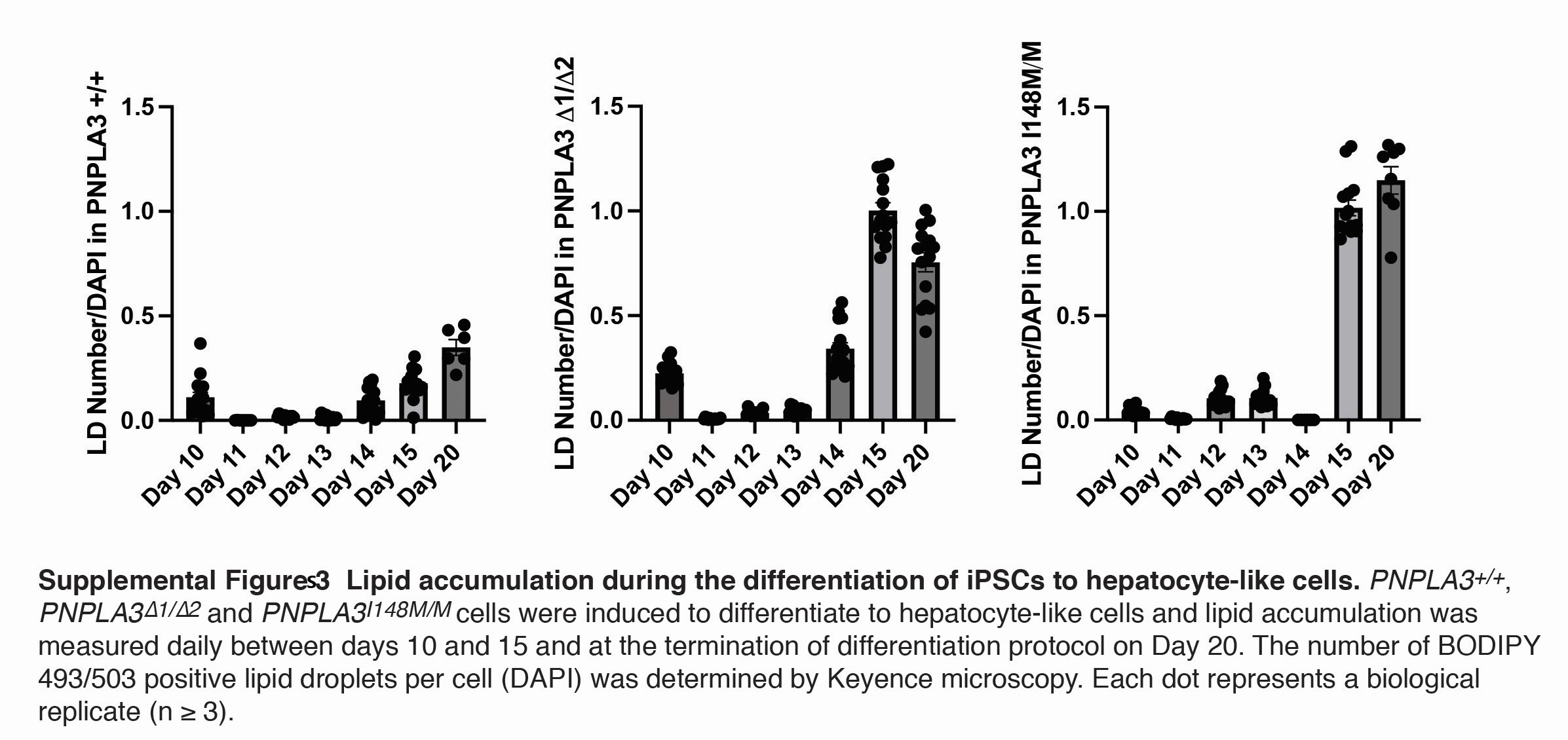

Supplement: Supplementary file 1 [file ijms-25-07277-s001.zip › Figure S3.jpg]

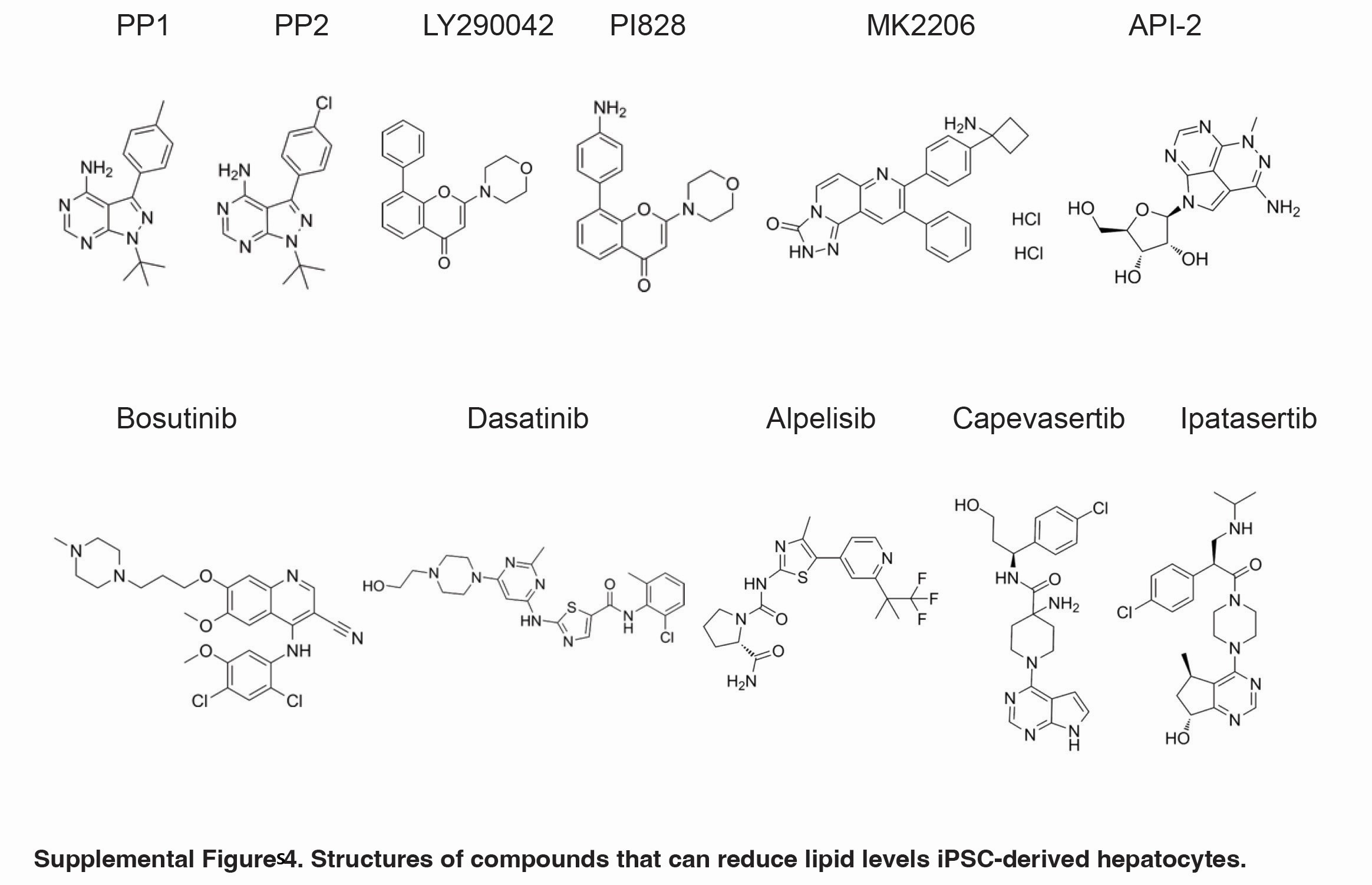

Supplement: Supplementary file 1 [file ijms-25-07277-s001.zip › Figure S4.jpg]

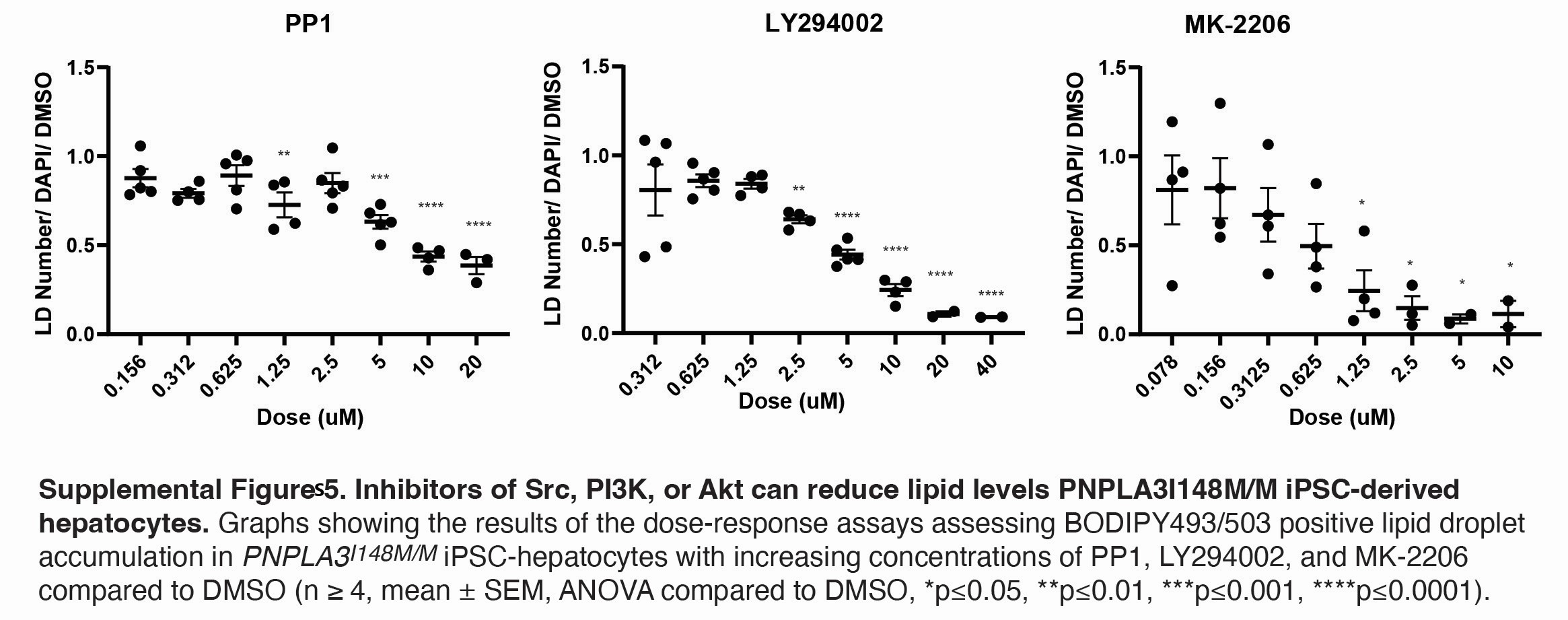

Supplement: Supplementary file 1 [file ijms-25-07277-s001.zip › Figure S5.jpg]

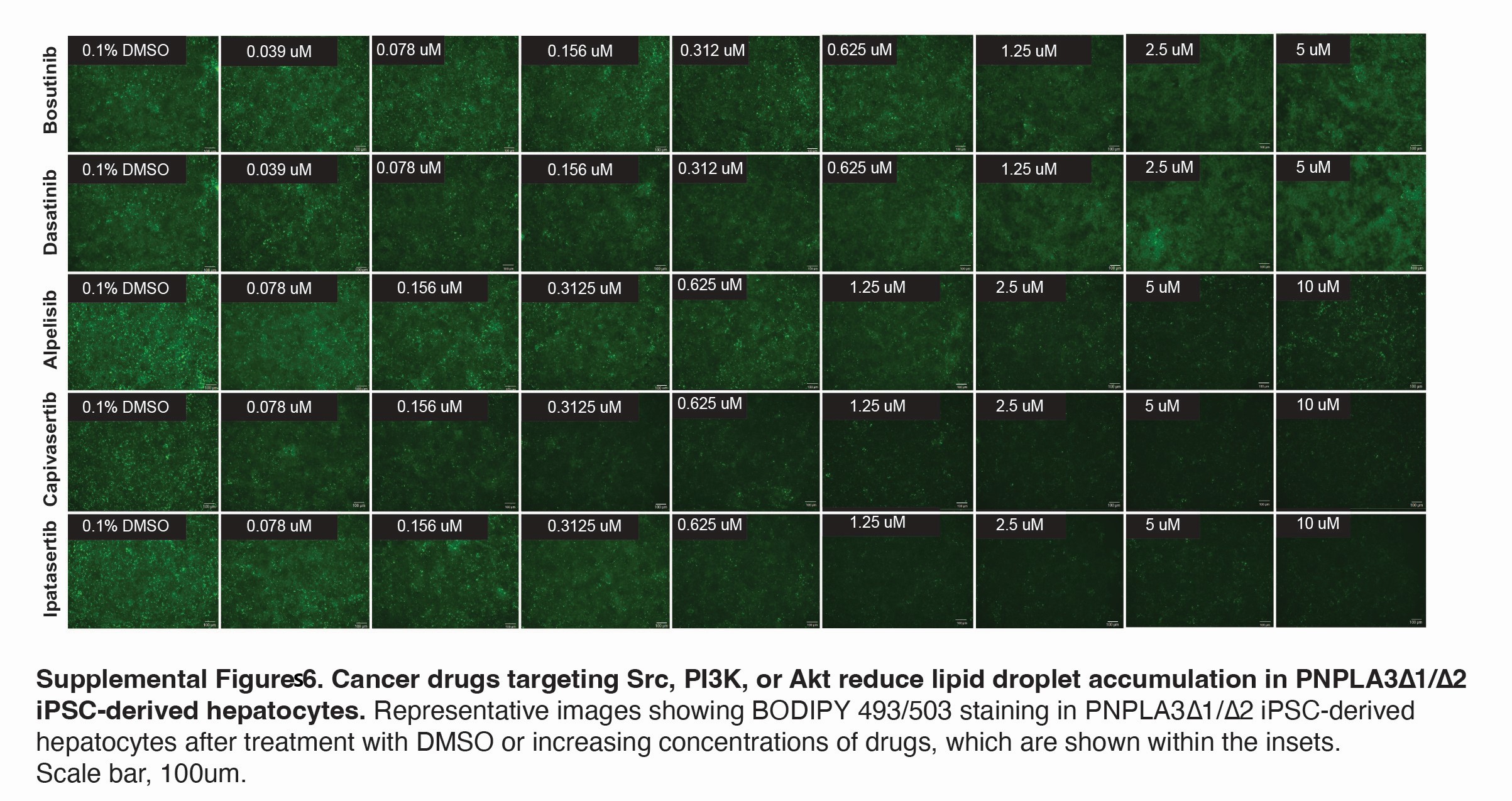

Supplement: Supplementary file 1 [file ijms-25-07277-s001.zip › Figure S6.jpg]
